# Connected care for endocarditis and heart failure patients: a hospital-at-home programme

**DOI:** 10.1007/s12471-021-01614-2

**Published:** 2021-09-15

**Authors:** J. van Ramshorst, M. Duffels, S. P. M de Boer, A. Bos-Schaap, O. Drexhage, S. Walburg, J. de Beij, D. van der Stoop, V. A. W. M. Umans

**Affiliations:** 1 Department of Cardiology, Noordwest Ziekenhuisgroep, Alkmaar, The Netherlands; 2 Omring, Hoorn, The Netherlands

**Keywords:** Endocarditis, Heart failure, Hospital at home, Antibiotic treatment

## Abstract

**Background:**

Healthcare expenditure in the Netherlands is increasing at such a rate that currently 1 in 7 employees are working in healthcare/curative care. Future increases in healthcare spending will be restricted, given that 10% of the country’s gross domestic product is spent on healthcare and the fact that there is a workforce shortage. Dutch healthcare consists of a curative sector (mostly hospitals) and nursing care at home. The two entities have separate national budgets (€25 bn + €20 bn respectively)

**Aim:**

In a proof of concept, we explored a new hospital-at-home model combining hospital cure and nursing home care budgets. This study tests the feasibility of (1) providing hospital care at home, (2) combining financial budgets, (3) increasing workforces by combining teams and (4) improving perspectives and increasing patient and staff satisfaction.

**Results:**

We tested the feasibility of combining the budgets of a teaching hospital and home care group for cardiology. The budgets were sufficient to hire three nurse practitioners who were trained to work together with 12 home care cardiovascular nurses to provide care in a hospital-at-home setting, including intravenous treatment. Subsequently, the hospital-at-home programme for endocarditis and heart failure treatment was developed and a virtual ward was built within the e‑patient record.

**Conclusion:**

The current model demonstrates a proof of concept for a hospital-at-home programme providing hospital-level curative care at home by merging hospital and home care nursing staff and budgets. From the clinical perspective, ambulatory intravenous antibiotic and diuretic treatment at home was effective in safely achieving a reduced length of stay of 847 days in endocarditis patients and 201 days in heart-failure-at-home patients. We call for further studies to facilitate combined home care and hospital cure budgets in cardiology to confirm this concept.

## What’s new?

- Combining the hospital cure and home care budgets by initiating collaboration between the Noordwest Ziekenhuisgroep and Omring facilitated a hospital-at-home programme for frail cardiology patients.
- The home care group budget was sufficient to hire three nurse practitioners who were trained by the cardiologists. The hospital budget was used to equip a virtual ward within the patient electronic record application.
- This doctor-driven transmural connected care model, combining the workforce and budgets of a teaching hospital and primary home care group, led to better Quadruple Aim outcomes.
- We safely achieved a reduced length of stay of 847 days in endocarditis patients and 201 days in heart-failure-at-home patients without unexpected major cardiovascular events.

## Introduction

In 2012, the Netherlands Society of Cardiology (NVVC) started a robust programme, NVVC Connect, to stimulate seamless care between hospital and primary care by connecting cardiologists with general practitioners and other primary care providers [1]. The main aim of this programme was to safely and expeditiously provide guideline-driven care for patients through optimal alignment of all the caregivers involved. It indeed embeds the concept of treating the patient at the best site and with the best care possible.

In 2018, the Dutch government set up a task force, *Juiste Zorg op de Juiste Plek *(Right Care in the Right Place), which developed a programme to relocate care, facilitate the use of e‑health applications and prevent more expensive care [2]. This programme also provides tools to support caregivers in building transmural networks allowing hospital care to be transformed into treatment at home.

The transition towards providing treatment at home started in the late 1990s, most notably by the Johns Hopkins group [3], focusing on frail elderly patients. With increasing experience, current European Society of Cardiology guidelines [4] provide opportunities and guidance on how to treat patients with endocarditis at home, while recent experience in heart failure treatment at home has also provided promising data [5–10].

Given that healthcare expenditure is increasing at such a rate that currently 1 in 7 Dutch employees are working in healthcare/curative care, an effort should be made to improve resource utilisation by building networks, relocating care and redesigning care processes. Therefore, we explored a new hospital-at-home model combining hospital cure and nursing home care budgets in an 800-km^2^ district with 650,000 inhabitants. The objective of this programme was to test the feasibility of (1) providing hospital care at home, (2) combining both financial budgets, (3) increasing workforces by combining teams and (4) improving perspectives and increasing the satisfaction of patients and nursing staff.

## Methods

### Connected care

**Fig. 1:**
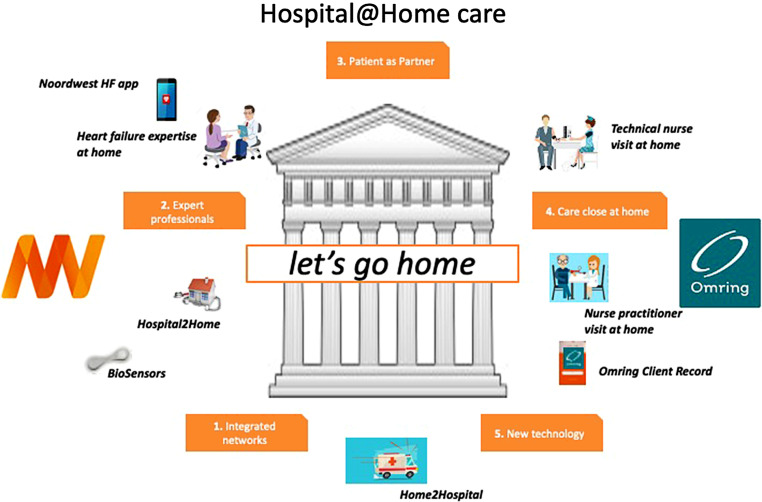
Organisational hospital-at-home model of the Noordwest Ziekenhuisgroep and Omring. On the *left*, the Noordwest activities and devices are depicted; on the *right* are those of Omring. Noordwest develops home-to-hospital protocols with mobile laboratory services and hospital-to-home protocols with Omring. Omring provides care at home with technical care nurses and nurse practitioners

Dutch healthcare consists of a cure sector (mostly hospitals) and a care sector, including nursing homes and home care providers, which are often united in larger VVT (*Verpleging Verzorging en Thuiszorg*; Nursing, Care and Home Care) institutions. Noordwest Ziekenhuisgroep and the VVT institution Omring started collaborating to develop a hospital-at-home programme for frail cardiology patients (Fig. 1). Following this board decision, staff and nursing working groups started connecting care workers and protocols. Directly involved staff were cardiologists, operations and project managers from both institutions, and the hospital pharmacist and discharge support group. The Information and Communications Technology departments of both organisations were involved for electronic health record (EHR) issues. The hospital EHR was adjusted to accommodate a hospital-at-home virtual ward.

The nursing staff may be considered the core developers. They are seven hospital-based nurse practitioners and 12 Omring technical care nurses. During several seminars, the implementation of a hospital at home was devised and logistic, medical and technical issues were addressed in working protocols. Meticulous attention was paid to patient safety and additional training of nurses. Finally, other relevant care providers, such as general practitioners and emergency medical services, were informed about the project.

The endocarditis-at-home protocols are merged with already existing local antibiotic-at-home treatments. These patients are discharged with central venous line antibiotic regimens with visits up to three times a day by home care nurses who replace the cassettes with antibiotics and perform central venous line management. All patients are seen once a week by the nurse practitioner at the outpatient clinic with (home) measurement of C‑reactive protein (CRP) levels and an echocardiographic examination when appropriate. Following termination of the antibiotic treatment at home, control CRP and blood cultures are performed. This date is considered the final day at home and the day of discharge from the virtual ward.

The heart-failure-at home protocols were specifically developed for frail [11] end-stage heart failure patients in need of intravenous (i.v.) diuretics twice a day.

The transfer and treatment-at-home protocols describe the hospital discharge and transfer of a patient around noon to the virtual ward with an afternoon visit at home by the Omring technical home care nurse to provide i.v. diuretic treatment. On all subsequent days, the patient is visited in the morning by the nurse practitioner and in the afternoon by the technical home care nurse. The nurse practitioner takes notes, performs physical examinations, provides i.v. treatment at home and prescribes appropriate doctor’s orders. The supervisory cardiologist is called once a day for feedback. The technical home care nurse provides the afternoon patient care and i.v. treatment at home. Out-of-office hours telephone standby is provided by the coronary care unit (CCU) nursing and medical staff. The general physician is on standby in case of a non-cardiac medical disorder.

The general practitioner is notified when endocarditis or heart failure patients have finished their i.v. treatment at home and are discharged from the virtual ward.

### Patients

The endocarditis cohort comprised admitted stable endocarditis patients who were treated for at least 14 days following a negative blood culture with culture-driven high-dose i.v. antibiotics through a central venous line. Eligible for endocarditis treatment at home were stable patients with a CRP level decreased to 20 mg/l, those with uncomplicated native or replaced biological valve endocarditis, postoperative endocarditis patients who have recently undergone valve surgery, patients with pacemaker lead endocarditis (following lead extraction) with or without an external lead, and end-stage patients without further therapeutic options. Local valve, conduction complications, uncontrolled infections or mechanical prosthesis endocarditis were exclusion criteria.

The heart failure cohort comprised frail elderly patients admitted for decompensated heart failure across the spectrum of ejection fraction. Following initial i.v. treatment, these patients were eligible for early discharge and treatment at home if further invasive diagnostics had been performed earlier and no malignant arrhythmias, inotropic dependence or severe renal dysfunction (MDRD < 30 ml/min) was present.

Throughout the first Covid lockdown, decompensated ambulatory patients in whom oral diuretic therapy had failed were also included. During this pilot, no patients were included on Fridays or at weekends.

Patients were subsequently scheduled for treatment at home twice a day by a registered nurse, as described above. Ambulatory laboratory or electrocardiographic controls were performed on indication. Patients were treated and followed until euvolaemia was reached. Finally, patients attended the outpatient heart failure clinic at 30 days.

### Outcomes

Evaluation of these preliminary results of connected care encompasses the full range of the Quadruple Aim. Patient and team satisfaction are established by interviews and questionnaires [12]. Surrogate outcome measures for costs are the admission days and car rental days saved.

The clinical efficacy outcomes in endocarditis at home are an uncomplicated clinical course during the remaining i.v. antibiotic treatment without local complications. Outcomes in heart failure at home include resolution of dyspnoea and/or oedema and weight loss. The evaluated safety outcomes included hypokalaemia and worsening renal function.

## Results

### Connected care

In a proof of concept, we tested the feasibility of combining the budgets of the hospital and home care group for cardiology. The home care budget allowed three new nurse practitioners to be hired who were trained by the cardiology group to work together with the home care cardiovascular nurses. In addition, these nurses played a pivotal role in the development of the programme. Furthermore, the budgets were sufficient to obtain laptops, mobile devices, rental cars for home visits and to employ supporting staff.

The hospital nurse practitioners provided on-the-job training for the three newly appointed nurse practitioners through their academic training at school. The home care provider Omring supplied a technical nursing team of 12 nurses and a daycare nursing team of 100 employees. Clinical protocols of both organisations were aligned and additional training for i.v. medications was performed. All home care nurses were trained to give i.v. diuretics during a few days on the clinical ward and all hospital nurse practitioners were trained in home visits by home care nurses.

The endocarditis-at-home programme started in September 2018 and has been running continuously since then. For the heart-failure-at-home programme, two pilot run-in periods of 6 and 12 weeks were used. Each participating heart failure patient was visited twice a day for i.v. treatment at home. Medication verification was performed by mailing a photo to the central Omring office. The cardiologist was consulted daily by phone and no home visits were needed.

### Endocarditis

From 18 September 2018 to 3 August 2020, a total of 60 patients who fulfilled the modified Duke criteria for definite endocarditis were discussed by the endocarditis team. Of these, 34 (57%) patients were enrolled for outpatient parenteral treatment in the hospital-at-home programme. Three of these were considered inoperable or were in need of supportive care at a nursing home.

The most frequent reasons for exclusion were an ongoing unstable sepsis course or local complications (*n* = 15), unwillingness or inability to give informed consent (*n* = 3), too many comorbidities (*n* = 5) or treatment with amoxicillin (*n* = 3; in need of 6 daily visits).

**Fig. 2:**
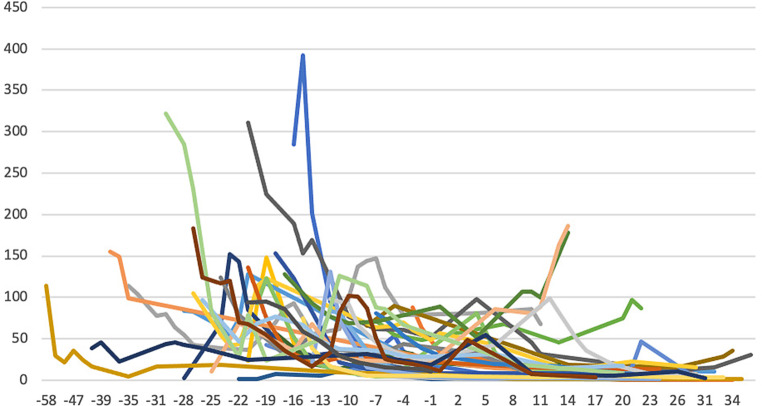
C‑reactive protein (CRP) levels during admission and treatment at home. CRP levels of all endocarditis-at-home patients since hospital admission. *Time* *=* *0* Admission to the virtual ward for intravenous antibiotic treatment ‘endocarditis at home’

The baseline characteristics of the patients are summarised in Tab. 1. The majority were male patients with a mean age of 70 ± 10 years. The native valve was affected in the majority of cases. The most frequently identified pathogen was *Streptococcus*. A total of 8 patients had an implanted cardiac device; 2 patients with pacemaker endocarditis had their pacemaker and leads removed during the current endocarditis disease course. The mean time from admission to starting outpatient treatment was 21 ± 10 days. After discharge, patients were treated at home according to the assigned regimen for a mean of 25 ± 10 days. A total of 847 hospital days were saved in these patients. CRP levels were followed on a weekly basis and are shown in Fig. 2.

**Table 1:** Baseline characteristics of endocarditis-at-home patients

Mean age (years)	70 ± 10
Female sex (%)	32
*Pathogens*	
*Streptococcus*	26
HACEK	1
*Staphylococcus*	8
Negative culture	3
Others	3
*Laboratory results*	
C-reactive protein (mg/l)	103
Creatinine (µmol/l)	116
Haemoglobulin (mmol/l)	7.3
Leucocytes (×10^−9^/l)	11.2
*Pre-existing prosthesis*	
Cardiac valve	8
Aortic prosthesis	1
Pacemaker/ICD	7
Non-cardiac	2
*Cardiac involvement*	
Native valve	18
Prosthetic valve	15
Aortic prosthesis	1
Pacemaker/ICD	6
Other cardiac location^a^	1

*ICD* implantable cardioverter-defibrillator

^a^Myoma

The number of outcome events during treatment at home is as follows. Two patients died, one (inoperable) suddenly due to an occlusive mitral prosthetic biovalve thrombus and one in a palliative home care centre. None of the patients experienced embolic episodes or relapse of bacteraemia with the primary pathogen. One patient required unplanned cardiac surgery. New significant CRP levels occurred in 5 patients and were all related to other infectious origins. The central venous line had to be changed in two patients.

### Heart failure

From March to December 2020, two pilots of 6 and 12 weeks were conducted. Ninety-five patients were admitted for acute heart failure treatment and lived in the Omring area. Of these, 12 (13%) patients were enrolled for outpatient parenteral treatment in the hospital-at-home programme. Two of these were in need of supportive care at a nursing home. Additionally, 4 derailed ambulatory patients were enrolled. Their demographics are displayed in Tab. 2.

**Table 2:** Baseline characteristics of heart-failure-at-home patients

Mean age (years)	76
Female sex (%)	8
Hypertension	9
Diabetes	3
Previous MI	6
Previous CABG/PCI	13
Atrial fibrillation	9
HFpEF	8
HFmrEF	4
HFrEF	4
Pacemaker	0
ICD	1

*MI* myocardial infarction, *CABG* coronary artery bypass grafting, *PCI* percutaneous coronary intervention, *HFpEF* heart failure with preserved ejection fraction, *HFmrEF* heart failure with mildly reduced ejection fraction, *HFrEF* heart failure with reduced ejection fraction, *ICD* implantable cardioverter-defibrillator

The most frequent reasons for exclusion were an early switch to oral therapy (*n* = 34) and the need for inotropy or invasive cardiac studies (*n* = 18).

These 16 patients, who received two discrete treatment home visits per day, were included in this pilot analysis. Their median age was 78 years (interquartile range 70–81 years). There were no gender differences and 50% of the patients experienced heart failure with preserved ejection fraction. At inclusion, all patients were elderly with end-stage heart failure class 3 and were moderately frail, with 3 being Clinical Frailty Scale class ‘vulnerable’. The mean admission length from onset of i.v. diuretics was 3 ± 2 days, and the mean duration of treatment at home was 11 ± 6 days, in total 201 days.

Mean clinic weight loss was 1.2 kg. Mean home weight loss was 6.3 kg. Home visits were performed twice a day in all cases, with an average patient burden of 3/day. On average, the patients lived 31 km (0.5–44 km) from the hospital. Mean travel time to the patient was 35 min. Patients were visited and treated at home on average 11.5 ± 5.2 days. Car rental was needed for 105 days.

During the home treatment phase, one hospitalisation occurred for i.v. inotropic treatment and none of the patients died. No patients needed to call the emergency CCU service, neither were there minor complications such as falls, delirium episodes or line infections. Renal and electrolyte measurements were all performed at home (Tab. 3) and a home-made electrocardiogram was needed once.

**Table 3:** Biomarkers during heart-failure-at-home (H@H) therapy

	Admission	H@H start	H@H end
Weight (kg)	89.0 ± 18.2	87.8 ± 17.5	81.5 ± 17.0
eGFR (ml/min)	59 ± 21	59 ± 19	51 ± 21
Urea (mmol/l)	9.1 ± 3.7	10.2 ± 5.6	11.9 ± 4.4
Sodium (mmol/l)	137 ± 5	138 ± 6	137 ± 4
Potassium (mmol/l)	4.1 ± 0.6	4.0 ± 0.6	4.1 ± 0.4

*eGFR* estimated glomerular filtration rate

At 30-day follow-up only one hospitalisation was necessary (a patient with heart failure); one nursing home care patient died.

### Patient and nurse satisfaction

Sixteen heart failure patients were eligible for telephone interviewing using the EQ-5D questionnaire at follow-up. Three patients were excluded: 2 who were living in the dementia department and 1 patient who died due to heart failure. Two (15%) of 13 patients refused to participate. All 11 patients rated the quality of care as good or excellent; they all received care at home as they had expected and which covered their needs. All patients would recommend heart-failure-at-home care to others and would choose this option again.

All of the technical care nurses and nurse practitioners (*n* = 16) involved completed the questionnaire and were satisfied (63%) or highly satisfied (25%) with their role, felt they obtained enough knowledge (88%), and 88% were (highly) satisfied with providing heart-failure-at-home care.

## Discussion

This doctor-driven transmural connected care model, combining the workforce and budgets of a teaching hospital and primary home care group led to an efficient hospital-at-home treatment programme for i.v. endocarditis and heart failure treatment at home with satisfactory Quadruple Aim outcomes. Working together led to an increase in nursing staff and patient satisfaction rates. From the clinical perspective, ambulatory i.v. antibiotic endocarditis and loop diuretic treatment at home using a standardised protocol is effective in safely achieving a mean reduced length of hospital stay of 847 and 201 days for endocarditis and heart failure, respectively.

### Connected care

The Dutch health system acknowledges four levels of care with ambulatory and institutional/home care being the base level involving the largest group of patients. Elderly frail patients who are followed or treated for chronic stable disease represent a substantial proportion of this population. These patients are treated mainly at home or in an elderly care institution. Typically, the patients are helped by level 3 nurses and in the case of intensive care by level 5 nurses. At the other extreme of this system are the teaching and university hospitals, which are equipped for acute and intensive care at expensive facilities. Care is typically provided by high-end medical specialists and level 5 nurses and nurse practitioners. We sought to combine workforces and budgets to cooperate along the lines of teaching and caring: caring for sicker patients in need of hospital admissions and teaching in guideline-provided care for home care nurses, thereby providing new working perspectives for nurses and staff. Hereby, we were able to form a group of nurses and nurse practitioners who were adequately trained and equipped to safely treat and care for hospital-at-home patients. Alongside this initiative, the Dutch Healthcare Authority (NZA) and the Federation of Medical Specialists (FMS) [2, 13] have developed models for connected care including financial incentives to facilitate transferring such care.

### CRP-driven antibiotic endocarditis-at-home treatment

This initial doctor-driven programme with treatment at home for endocarditis patients provides insights into safe care at home, thereby reducing the length of stay by 25 days on average. This endocarditis-at-home option is available for stable patients on antibiotics, for frail elderly patients in whom no further options are available and for stable postoperative patients. These results are comparable with those of the Danish trial on oral continuation of treatment [14]. Additionally, we extend the experiences described in the guidelines [4, 15–18], where home treatment is limited to patients without prosthetic valves or *Staphylococcus aureus* infections. Our data suggest that once the CRP levels in clinically stable patients are decreasing steadily, patients across a variety of endocarditis causes may be eligible for outpatient parenteral treatment. The main focus should, however, be on unlimited direct access to the endocarditis team in case of clinical worsening. In our experience, the guidance on weekly CRP levels prevented such immediate consultations. Treatment at home had a major impact on patient well-being, with none of them regretting haven given their consent. Their family members did not need to travel for hospital visits and some weekly visits were rescheduled as (video)teleconferencing visits during the COVID pandemic.

### Decongestion-driven i.v. heart failure at home

Decompensated heart failure with fluid overload remains an important cause of frequent hospital admissions for i.v. diuretic therapy. A previous retrospective study found outpatient i.v. diuretic therapy to be safe and effective [7, 19]. Our pilot study examines an innovative way of providing i.v. diuretic treatment at home in an effective manner in an outpatient setting with a reduced length of hospital stay. The regimen was safe in that it did not cause any significant drop in blood pressure or unexpected derangement of renal function or electrolytes. Hospital readmission for inotropy was needed in one patient. The financial gain as a result of reducing length of hospital stay was substantial. In addition, since hospital at home allows home-based interventions and provides continuity of patient care beyond the conventional ambulatory settings [20–23], it is conceivable that the frequency of unplanned readmissions in heart failure patients will decrease. Seen in perspective, these hospital-at-home outcome observations are important because they provide a ‘blueprint’ for reducing length of hospital stay and potentially reducing heart failure hospitalisations. Finally, in the future it may be possible to start i.v. diuretic treatment directly at home by transmural consultation, without any hospital visit. Since a significant number of patients in our cohort could be switched rapidly from i.v. to oral diuretics this may be effective in preventing readmissions.

## Conclusion

This doctor-driven transmural connected care model, combining the workforce and budgets of a teaching hospital and primary home care group led to better Quadruple Aim outcomes: efficient hospital-at-home treatment programmes for i.v. endocarditis and heart failure treatment. Working together led to increased nursing staff and improved patient perspectives. From the clinical perspective, ambulatory i.v. antibiotic and diuretic treatment at home is effective in safely achieving a reduced length of stay of 847 days in endocarditis patients and 201 days in heart-failure-at-home patients without unexpected major cardiovascular events.
